# Young adult polysubstance use trajectories and later non-prescribed opioid use and drug use disorder symptoms: A propensity score-matched analysis

**DOI:** 10.1016/j.dadr.2026.100479

**Published:** 2026-09-12

**Authors:** Moriah R. Harton, Jon Agley, Dong-Chul Seo, Roger S. Zoh, Maria A. Parker

**Affiliations:** a Indiana University Bloomington, School of Public Health, Department of Epidemiology and Biostatistics, 1025 E. 7th St, Bloomington, IN 47405, United States; b Indiana University Bloomington, School of Public Health, Department of Applied Health Science, 1025 E. 7th St, Bloomington, IN 47405, United States

**Keywords:** Polysubstance use, Non-prescribed opioid use, Substance use trajectories, Group-based trajectory modeling, Propensity score analysis, Young adulthood, Monitoring the Future

## Abstract

**Background:**

Longitudinal patterns of common substance use in young adulthood may identify risk for later opioid-related and other drug-related harm. We examined whether longitudinal trajectories of alcohol, cannabis, and nicotine use during young adulthood were associated with non-prescribed opioid use and other drug use disorder (ODUD) symptoms in adulthood.

**Methods:**

We analyzed restricted longitudinal Monitoring the Future panel data from 26 baseline cohorts (1976–2002). Group-based trajectory modeling identified polysubstance use trajectories from baseline through the first three biennial follow-ups. Trajectories were classified as low-use or high-use. Outcomes were any non-prescribed opioid use and ODUD symptoms between ages 35 and 55. Propensity score weighting estimated associations under average treatment effect for the treated, average treatment effect for the untreated, and average treatment effect estimands.

**Results:**

Five trajectory groups were identified and classified as low-use or high-use. The low-use trajectory included 14.7% of participants and was characterized by no cannabis or nicotine use and low-to-moderate alcohol use; the remaining trajectories involved high use of at least one substance. In average treatment effect models, high-use trajectory membership was associated with higher odds of later non-prescribed opioid use (OR=3.61; 95% CI: 3.34, 3.91) and ODUD symptoms (OR=15.9; 95% CI: 13.5, 18.8).

**Conclusion:**

In this US national longitudinal cohort, young adults with sustained high use of alcohol, cannabis, and/or nicotine had higher odds of non-prescribed opioid use and ODUD symptoms later in adulthood. These findings support targeted prevention, harm reduction, and cessation efforts addressing polysubstance use among young adults.

## Highlights

• Five young adult polysubstance use trajectories were identified.
• High-use trajectories predicted later non-prescribed opioid use.
• High-use trajectories predicted adult drug use disorder symptoms.
• Associations persisted after propensity score weighting.

## 1 Introduction

Young adulthood is an important developmental period for understanding how substance use patterns relate to later drug-related harm. In the United States, non-prescribed opioid use and opioid-related morbidity have remained major public health concerns for more than two decades, yet the developmental pathways leading to these outcomes are not fully understood (Blanco and Volkow, 2019, CDC, 2011, Compton et al., 2019, Mattson et al., 2021). Although the epidemiology of non-prescribed opioid use and opioid use disorder has been well described across a range of populations, less is known about how early patterns of use involving more common substances may contribute to later opioid-related outcomes (Han et al., 2018, Han et al., 2017, Hudgins et al., 2019, John et al., 2019, Olfson et al., 2023).

Use of alcohol, cannabis and nicotine is prevalent in young adulthood; these substances are commonly co-used and their use is potentially modifiable through prevention and early intervention (Cicero et al., 2020, Compton et al., 2019, Crummy et al., 2020, Richter et al., 2017). Previous research has shown that using any of these substances is associated with non-prescribed opioid use and opioid use disorder, though polysubstance use has been increasingly recognized as a central feature of drug-related harm (Chou et al., 2016, Cicero et al., 2020, Compton et al., 2019, Mahoney et al., 2021, Olfson et al., 2023, Parker and Weinberger, 2020, Votaw et al., 2019). Much of the existing literature on substance use has focused on single substances, cross-sectional associations or later stage drug-related harms, leaving uncertainty about whether distinct longitudinal trajectories of alcohol, cannabis and nicotine use in young adulthood are associated with opioid-related outcomes later in life (Huang et al., 2019, Kwon et al., 2021, Lee et al., 2022, McCabe et al., 2019b). Prior longitudinal work has linked alcohol use trajectories with subsequent opioid misuse, and a systematic review and meta-analysis found that cannabis use was associated with later opioid use and opioid related disorder outcomes (Thrul et al., 2021, Wilson et al., 2022).

Patterns of substance use in young adulthood are heterogeneous. Some individuals show persistently low use, whereas others exhibit sustained or escalating use of one or more substances. Identifying these trajectories may help clarify whether prolonged high-use patterns serve as a meaningful pathway to later harm (Kwon et al., 2021, McCabe et al., 2019b, McCabe et al., 2022a). This question is especially important for common, legal, and/or widely accessible substances such as alcohol, cannabis and nicotine, because even modest increases in opioid-related risk could have considerable public health implications (Cicero et al., 2020, Compton et al., 2019, Crummy et al., 2020).

The Monitoring the Future (MTF) panel study has been widely used to study developmental patterns of substance use and later substance-related outcomes, including trajectories of prescription drug misuse from ages 18–50, adolescent substance use disorder symptoms and later adult substance use disorder, and associations between adolescent nonmedical prescription opioid use and later substance use disorder symptoms (McCabe et al., 2019b, McCabe et al., 2022a, McCabe et al., 2022c). These studies demonstrate the utility of MTF panel data for examining long-term associations between early substance-use patterns and later drug-related outcomes. Related MTF work has identified young adult polysubstance use trajectories associated with non-prescribed opioid use during the same young adult developmental period (Harton and Parker, 2027).

In addition to non-prescribed opioid use, drug-related morbidity beyond opioid use disorder warrants attention. In longitudinal survey data, it is often difficult to isolate opioid use disorder diagnoses with precision over extended follow-up periods because diagnostic information is limited, substance-specific symptom attribution can be incomplete, and symptoms may be assessed across broad follow-up intervals (McCabe et al., 2022a, Tang et al., 2024). For this reason, other drug use disorder (ODUD) symptoms may serve as a complementary indicator of later drug-related harm, capturing the impact of problematic substance use in adulthood (McCabe et al., 2019b, McCabe et al., 2019a, McCabe et al., 2022a, McCabe et al., 2022c). Therefore, methodologically, examining both non-prescribed opioid use and ODUD symptoms is a way to determine whether young adult polysubstance use trajectories are associated with later opioid use and with subsequent drug use disorder symptoms in adulthood.

Estimating these associations requires careful attention to baseline differences between individuals with different substance use patterns (Grigoras et al., 2018, Olfson et al., 2023, Romeiser et al., 2019, Schuler et al., 2020, Serdarevic et al., 2017). Longitudinal observational data, combined with trajectory modeling and propensity score methods, offer an opportunity to examine whether observed associations are consistent with a possible causal relationship (Nagin, 2005, Nagin et al., 2024, Nguena Nguefack et al., 2020, Rosenbaum and Rubin, 1983).

Using nationally representative data from the MTF panel study, we examined whether trajectories of alcohol, cannabis and nicotine use from ages 18–24 were associated with later non-prescribed opioid use and ODUD symptoms between ages 35 and 55. (Patrick et al., 2022, Schulenberg et al., 2022). We identified distinct polysubstance use trajectories using group-based trajectory modeling and then used propensity score weighting to estimate associations between membership in high-use trajectory groups and subsequent adult outcomes. We hypothesized that membership in high-use young adult polysubstance trajectories would be associated with increased odds of both non-prescribed opioid use and ODUD symptoms in later adulthood.

## 2 Methods

### 2.1 Study design and data source

We used restricted longitudinal panel data from the MTF study, an ongoing nationally representative cohort study of substance use and related behaviors in the United States. Each year, a nationally representative sample of 12th-grade students is selected using a multistage school based sampling design, and a subsample is selected for longitudinal panel follow-up. Since 1976, each annual cohort has contributed a semi-random subsample of approximately 2450 individuals to panel follow-up, with oversampling of participants with substance use. The MTF panel follows these participants beginning at modal age 18, with follow-up surveys administered biennially through age 30 and every 5 years thereafter (Patrick et al., 2022, Schulenberg et al., 2022). At each wave, participants complete self-administered questionnaires assessing substance use, health behaviors, educational and social characteristics, and other developmental outcomes. Because MTF is school based at baseline, adolescents who were not enrolled in school at the time of sampling are not represented in the original cohort.

For the present study, we examined whether trajectories of alcohol, cannabis and nicotine use from age 18 through young adulthood were associated with later non-prescribed opioid use and ODUD symptoms in adulthood. The analysis used data from respondents who completed the baseline survey, at least one of the first three follow-up surveys, and at least one follow-up survey between ages 35 and 55. This secondary analysis of restricted-use deidentified MTF data was reviewed by the Indiana University - Bloomington institutional review board and determined to be non-human subjects research (protocol #16479).

### 2.2 Study sample

Participants were first eligible if they completed the baseline survey and at least one of the first three follow-up surveys at modal ages 19/20, 21/22 and 23/24, and provided data on alcohol, cannabis and nicotine use at baseline. Of 103,939 participants in the original sample, 19,407 were excluded because of missing exposure-related measures, yielding 84,307 participants eligible for trajectory classification.

Two adult outcome specific samples were then contstructed separately. For the non-prescribed opioid use analysis, we excluded participants with complete missingness on the outcome during follow-up between ages 35 and 55 and those reporting non-prescribed opioid use at baseline, resulting in an analytic sample of 28,924 participants drawn from 26 baseline cohorts (1976–2002). Adult outcome observations between modal ages 35 and 55 corresponded to calendar years 1993–2019, depending on cohort and follow-up age. For the ODUD symptoms analysis, participants with complete missingness on the symptom outcome during follow-up were excluded, resulting in an analytic sample of 28,275 participants from the same 26 baseline cohorts. Participants with missing exposure measures or complete missingness on the relevant outcome were excluded; analyses were otherwise conducted on complete cases within each analytic sample.

## 3 Measures

### 3.1 Exposure: young adult polysubstance use trajectories

Alcohol, cannabis and nicotine use were measured from baseline through the first three biennial follow-up waves, corresponding approximately to ages 18 through 23/24. Use of each substance was dichotomized at each wave as “use” versus “no use” during the past 12 months, except for baseline nicotine use, which was coded as “ever” versus “never” use because of differences in available survey items. The exposure indicators were based on self-reported use of: (1) alcohol, (2) cannabis, and (3) nicotine. Specifically, exposure was based on self-reported past-year alcohol use (“On how many occasions [if any] have you had any alcoholic beverage to drink—more than just a few sips in the past 12 months?”), past-year cannabis use (“On how many occasions [if any] have you used marijuana [weed, pot] or hashish [hash, hash oil] in the past 12 months?”), and baseline ever cigarette use (“Have you ever smoked cigarettes?”).

Group-based trajectory modeling (GBTM) was used to identify distinct longitudinal patterns of alcohol, cannabis and nicotine use across young adulthood (Nagin, 2005, Nagin et al., 2024). GBTM is a finite mixture approach that identifies subgroups of individuals with similar developmental patterns over time and assumes discrete latent trajectory groups with no within class variation. After trajectory groups were identified, groups characterized by high use of at least one substance were classified as high-use, whereas the group characterized by no cannabis use, no nicotine use and low-to-moderate alcohol use was classified as low-use and served as the reference group in subsequent analyses.

### 3.2 Outcomes

Two adult outcomes were examined: non-prescribed opioid use and ODUD symptoms between ages 35 and 55.

### 3.3 Non-prescribed opioid use

The first outcome was any non-prescribed opioid use reported between ages 35 and 55. This was defined as any past-year use of heroin and/or ‘narcotics other than heroin’ without a doctor telling the participant to take them. Participants were classified as having the outcome if they reported either form of non-prescribed opioid use at any follow-up wave during this age range. This outcome was based on self-reported past-year use of heroin (“On how many occasions [if any] have you taken heroin in the last 12 months?”) and narcotics other than heroin (“On how many occasions [if any] have you taken narcotics other than heroin—that is, without a doctor telling you to take them in the past 12 months?”).

### 3.4 Other drug use disorder (ODUD) symptoms

The second outcome was ODUD symptoms between ages 35 and 55. This outcome was derived from 15 survey items corresponding to 8 DSM-5 substance use disorder criteria: failure to fulfill major role obligations, hazardous use, continued use despite social or interpersonal problems, tolerance, withdrawal, unsuccessful efforts to cut down, health-related problems due to use and craving (American Psychiatric Association, 2013). These items were used to operationalize symptom presence rather than clinical diagnosis in the available longitudinal survey data. Participants were classified as having ODUD symptoms if they reported two or more simultaneous symptoms during follow-up between ages 35 and 55. Details of the 15 MTF survey items used to derive the ODUD symptom indicator, their DSM-5 criterion mapping, and coding are provided in Supplemental Table 1. Although this measure does not represent a clinical diagnosis, it captures the impact of later drug-related symptoms in adulthood and is consistent with prior MTF research using substance use disorder symptom measures (McCabe et al., 2022b).

### 3.5 Covariates

Baseline covariates were selected a priori based on prior literature on substance use and opioid-related risk and included sex (male, female), race/ethnicity (Non-Hispanic White, Non-Hispanic Black, Hispanic, Asian, Other, Unknown), mother’s educational attainment (less than high school, high school graduate, some college, college graduate, graduate school), geographic location (Midwest, Northeast, South, West), urbanicity classification (rural, small town, suburban, urban, unknown), number of parents in household (two, one, zero/unknown), and GPA (A, B, C/D/Unknown) (Cornelius et al., 2023, Grigoras et al., 2018, Kurani et al., 2020, Olfson et al., 2023, Romeiser et al., 2019, U.S. Department of Health and Human Services, 2012, U.S. Department of Health and Human Services, 2000). Covariates were measured at baseline and were included in the propensity score models used to balance measured baseline covariates for each trajectory-group comparison.

### 3.6 Statistical analysis

#### 3.6.1 Group-based trajectory modeling (GBTM)

We used GBTM to identify subgroups of individuals with similar patterns of alcohol, cannabis, and nicotine use across the four young adult waves (Nagin, 2005, Nagin et al., 2024, Nguena Nguefack et al., 2020). Model selection proceeded in two stages. First, we fit models with between one and five groups using intercept only specifications. Candidate models were evaluated using the Bayesian information criterion (BIC), entropy, average posterior probabilities and odds of correct classification. Models were considered acceptable if entropy exceeded 0.80, average posterior probabilities for assigned groups exceeded 0.70, and odds of correct classification exceeded 5 for each group. The model with the BIC closest to zero among those meeting classification criteria was selected.

Second, conditional on the selected number of groups, we determined the polynomial order for each substance specific trajectory component by comparing intercept, linear, quadratic and cubic specifications and selecting the factorial combination with the BIC closest to zero (Kwon et al., 2021, Nagin, 2005, Nagin et al., 2024). Baseline characteristics across resulting trajectory groups were compared using chi-square tests. Trajectory analyses were conducted in Stata 18, accounting for the complex sampling design using Taylor series linearization for variance estimation (Stata Corp, 2021).

#### 3.6.2 Propensity score weighting

To examine whether the association between high-use young adult trajectories and later outcomes was consistent with a causal interpretation under no unmeasured-confounding assumptions, we estimated propensity scores for membership in the high-use trajectory group using logistic regression (Rosenbaum and Rubin, 1983). The propensity score model included all baseline covariates listed above. Separate propensity score analyses were conducted for each adult outcome sample. Propensity scores were estimated separately for each comparison between the low-use trajectory group and each of the other trajectory groups.

Using these estimated propensity scores, we constructed inverse probability weights for three estimands: the average treatment effect for the treated (ATT) [those with membership in high-use trajectory groups], the average treatment effect for the untreated (ATU) [those with membership in low-use trajectory groups], and the average treatment effect (ATE) [averaged over the sample population]. ATT estimates the association among participants whose observed trajectories were classified as high-use; ATU estimates the association if participants in the low-use group had instead followed high-use trajectories; and ATE estimates the average association in the combined analytic sample. Covariate balance before and after weighting was assessed using standardized mean differences, with values below 0.05 taken to indicate adequate balance. Propensity score overlap between the high-use and low-use groups was assessed graphically.

Extreme weights were observed for the ATT and ATE estimands. To reduce the influence of highly weighted observations, weights were truncated at the 99th percentile, such that values above the 99th percentile were set equal to the 99th percentile value (Stürmer et al., 2021).

#### 3.6.3 Outcome models

For each outcome, weighted logistic regression models were used to estimate the association between membership in the high-use trajectory group and adult non-prescribed opioid use or ODUD symptoms. Models were fit separately using ATT, ATU and ATE weights. Results are reported as odds ratios (ORs) with 95% confidence intervals (CIs). Propensity score estimation, weighting and outcome modeling were conducted in R version 4.4.3.

## 4 Results

### 4.1 Polysubstance use trajectories

Five distinct trajectories of alcohol, cannabis and nicotine use across young adulthood were identified. The selected five group solution met prespecified model fit and classification criteria based on the BIC, entropy, posterior probabilities, and odds of correct classification. Group 1, characterized by no cannabis use, no nicotine use and low-to-moderate alcohol use, included 9153 participants (14.7%) and served as the “low-use” reference group. The remaining four groups were classified as “high-use” because they were characterized by high use of at least one substance. Group 2 showed little to no cannabis or nicotine use with high alcohol use [20,351 participants (32.7%)]. Group 3 showed consistently high probabilities of alcohol, cannabis, and nicotine use across young adulthood [13,199 (21.2%)]. Group 4 showed low cannabis use, moderate-to-high nicotine use, and consistently high alcohol use [12,205 (19.6%)]. Group 5, showed high-to-moderate cannabis use, nicotine use that decreased from moderate levels across follow up, and high alcohol use [7315 (11.8%)] (Supplemental Figure 1).

For the non-prescribed opioid use analysis, 25,492 participants were classified in the high-use groups and 3432 in the low-use group. For the ODUD symptoms analysis, 24,932 participants were classified in the high-use groups and 3343 in the low-use group (Supplemental Table 2).

### 4.2 Sample characteristics

The two analytic samples were highly similar and are, therefore, described together. In both samples, 43% of participants were male and approximately 82% were non-Hispanic White. Compared with the low-use group, participants in the high-use groups were more likely to be male, non-Hispanic White, to live in suburban or urban areas rather than rural areas, and to reside in the Northeast or Midwest rather than the South (all p < 0.001). Maternal education as well as number of parents in household varied minimally between the overall samples and the high-use and low-use trajectory groups. The average GPA for both overall samples was a “B” while the high-use trajectory groups had significantly fewer participants with an “A” average GPA and significantly more participants with an “C/D/unknown” average GPA compared to the low-use groups (p < 0.001) (Table 1).

**Table 1:** Baseline characteristics of participants, overall and stratified high-use vs. low-use groups for the outcomes of non-prescribed opioid use and ODUD symptoms. Data from the Monitoring the Future Study, 1976-2019.

	Non-Prescribed Opioid Use				ODUD Symptoms			
	Overall	High-Use	Low-Use	p-value	Overall	High Use	Low-Use	p-value
	N = 28,924	N = 25,492	N = 3432		N = 28,275	N = 24,932	N = 3343	
*Sex*				< 0.001				< 0.001
Male	12,313 (43%)	10,975 (43%)	1338 (39%)		12,069 (43%)	10,766 (43%)	1303 (39%)	
Female	16,611 (57%)	14,517 (57%)	2094 (61%)		16,206 (57%)	14,166 (57%)	2040 (61%)	
*Race*				< 0.001				< 0.001
White	23,818 (82%)	21,409 (84%)	2409 (70%)		23,308 (82%)	20,955 (84%)	2353 (70%)	
Black	2089 (7.2%)	1592 (6.2%)	497 (14%)		2046 (7.2%)	1560 (6.3%)	486 (15%)	
Asian	611 (2.1%)	460 (1.8%)	151 (4.4%)		588 (2.1%)	444 (1.8%)	144 (4.3%)	
Hispanic	1326 (4.6%)	1121 (4.4%)	205 (6.0%)		1284 (4.5%)	1086 (4.4%)	198 (5.9%)	
Other	854 (3.0%)	716 (2.8%)	138 (4.0%)		829 (2.9%)	697 (2.8%)	132 (3.9%)	
Unknown	226 (0.8%)	194 (0.8%)	32 (0.9%)		220 (0.8%)	190 (0.8%)	30 (0.9%)	
*Urbanicity*				< 0.001				< 0.001
Rural	5818 (20%)	4945 (19%)	873 (25%)		5718 (20%)	4861 (19%)	857 (26%)	
Small Town	8654 (30%)	7690 (30%)	964 (28%)		8481 (30%)	7540 (30%)	941 (28%)	
Suburban	5794 (20%)	5252 (21%)	542 (16%)		5661 (20%)	5132 (21%)	529 (16%)	
Urban	6761 (23%)	5980 (23%)	781 (23%)		6572 (23%)	5817 (23%)	755 (23%)	
Unknown	1897 (6.6%)	1625 (6.4%)	272 (7.9%)		1843 (6.5%)	1582 (6.3%)	261 (7.8%)	
*Census Region*				< 0.001				< 0.001
South	8621 (30%)	7157 (28%)	1464 (43%)		8404 (30%)	6972 (28%)	1432 (43%)	
Northeast	6266 (22%)	5877 (23%)	389 (11%)		6137 (22%)	5754 (23%)	383 (11%)	
Midwest	9192 (32%)	8403 (33%)	789 (23%)		9020 (32%)	8253 (33%)	767 (23%)	
West	4845 (17%)	4055 (16%)	790 (23%)		4714 (17%)	3953 (16%)	761 (23%)	
*Mother’s Highest Educ.*				< 0.001				< 0.001
Less than High School	3986 (14%)	3448 (14%)	538 (16%)		3898 (14%)	3372 (14%)	526 (16%)	
High School Grad	10,751 (37%)	9533 (37%)	1218 (35%)		10,560 (37%)	9368 (38%)	1192 (36%)	
Some College	5236 (18%)	4605 (18%)	631 (18%)		5114 (18%)	4500 (18%)	614 (18%)	
College Grad	5385 (19%)	4760 (19%)	625 (18%)		5243 (19%)	4634 (19%)	609 (18%)	
Grad School	2591 (9.0%)	2336 (9.2%)	255 (7.4%)		2505 (8.9%)	2264 (9.1%)	241 (7.2%)	
Unknown	975 (3.4%)	810 (3.2%)	165 (4.8%)		955 (3.4%)	794 (3.2%)	161 (4.8%)	
*Num. Parents in Home*				> 0.9				0.9
2	23,034 (80%)	20,301 (80%)	2733 (80%)		22,525 (80%)	19,856 (80%)	2669 (80%)	
1	4694 (16%)	4141 (16%)	553 (16%)		4589 (16%)	4055 (16%)	534 (16%)	
0/Unknown	1196 (4.1%)	1050 (4.1%)	146 (4.3%)		1161 (4.1%)	1021 (4.1%)	140 (4.2%)	
*GPA*				< 0.001				< 0.001
A	8071 (28%)	6747 (26%)	1324 (39%)		7874 (28%)	6587 (26%)	1287 (38%)	
B	14,837 (51%)	13,261 (52%)	1576 (46%)		14,509 (51%)	12,976 (52%)	1533 (46%)	
C/D/Unknown	6016 (21%)	5484 (22%)	532 (16%)		5892 (21%)	5369 (22%)	523 (16%)	

### 4.3 Propensity score models and covariate balance

Before weighting, baseline covariates differed substantially between the high-use and low-use groups in both analytic samples. In the propensity score models, females had lower odds of membership in the high-use groups than males, and all non-White race/ethnicity groups had lower odds of high-use group membership compared with non-Hispanic White participants. Participants living in rural areas had lower odds of high-use group membership than those living in small towns, whereas participants living in suburban and urban areas had higher odds. Compared with participants living in the South, those living in the Northeast and Midwest had higher odds of membership in the high-use groups, as did those living in single-parent households and participants with lower grade point averages. Full model estimates are shown in Supplemental Table 1.

**Fig. 1:**
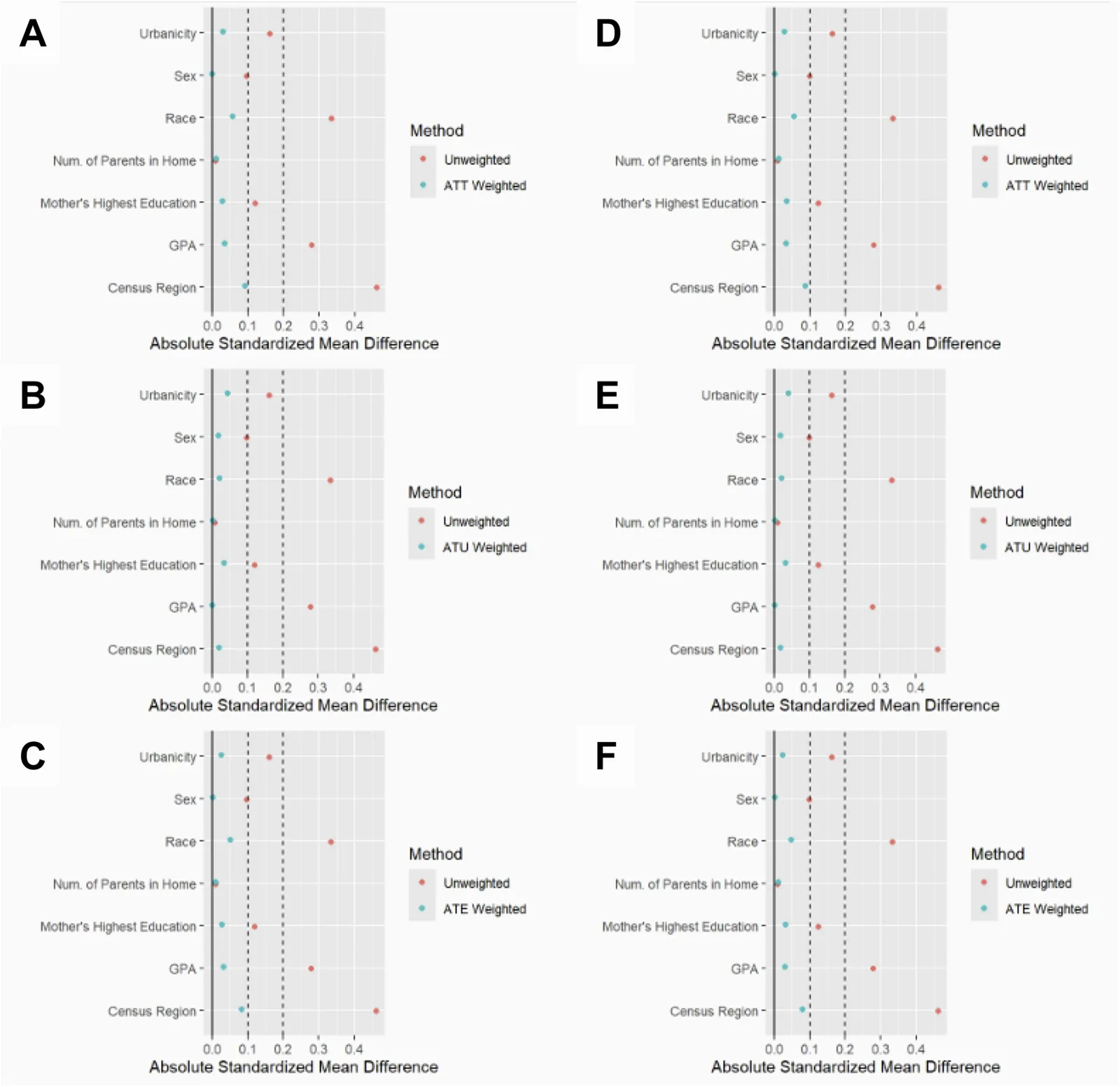
Love plots displaying covariate balance pre- and post-weighting for non-prescribed opioid use (A, B, C) and ODUD symptoms (D, E, F) using ATT weighting (A, D), ATU weighting (B, E), and ATE weighting (C, F). Data from the Monitoring the Future Study, 1976-2019.

**Fig. 2:**
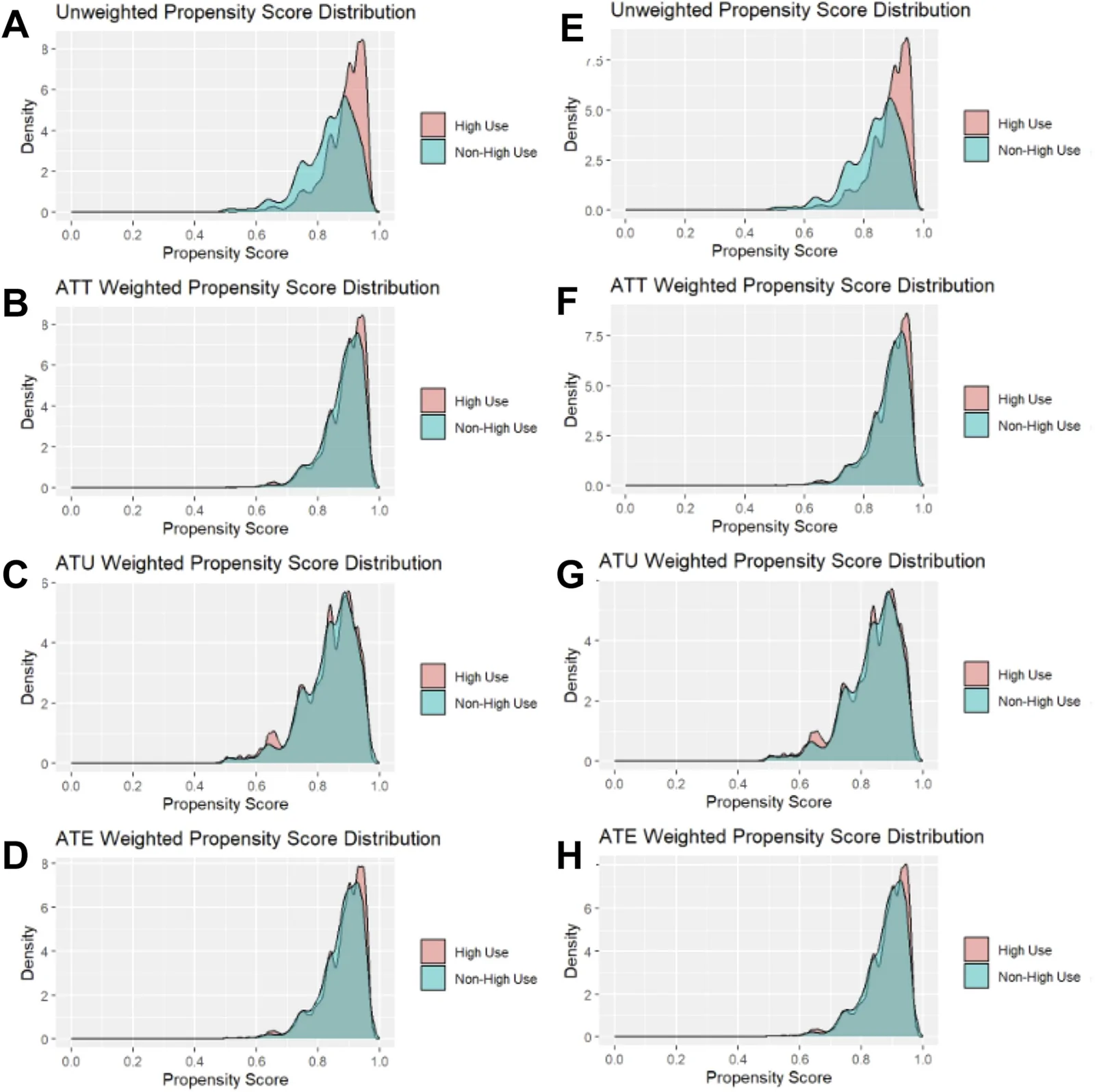
Propensity score density plots for non-prescribed opioid use (A, B, C, D) and ODUD symptoms (E, F, G, H). Density plots are unweighted (A, E), ATT weighted (B, F), ATU weighted (C, G), and ATE weighted (D, H). Data from the Monitoring the Future Study, 1976-2019.

After weighting, covariate balance improved for all estimands and both outcomes. All standardized mean differences were below 0.05 after weighting, indicating good balance between the high-use and low-use groups (Fig. 1). Propensity score distributions also showed adequate overlap before weighting and similar weighted distributions across groups after weighting (Fig. 2). Extreme weights were observed for ATT and ATE models and were truncated at the 99th percentile.

### 4.4 Non-prescribed opioid use

Membership in a high-use young adult polysubstance trajectory group was associated with higher odds of later non-prescribed opioid use across all weighting approaches. In the ATT-weighted model, OR= 3.67; 95% CI: 3.38, 4.00; p < 0.001. In the ATU-weighted model, OR= 3.17; 95% CI: 2.54, 3.98; p < 0.001. In the ATE-weighted model, OR= 3.61; 95% CI: 3.34, 3.91; p < 0.001 (Table 2).

**Table 2:** Odds of prospective non-prescribed opioid use and ODUD symptoms. Data from the Monitoring the Future Study, 1976-2019.

	Non-Prescribed Opioid Use		ODUD Symptoms		
	OR (95% CI)	p-value		OR (95% CI)	p-value
*ATT-weighted*					
High-Use Group	3.67 (3.38, 4.00)	< 0.001		16.4 (13.7, 19.7)	< 0.001
*ATU-weighted*					
High-Use Group	3.17 (2.54, 3.98)	< 0.001		12.8 (8.42, 20.6)	< 0.001
*ATE-weighted*					
High-Use Group	3.61 (3.34, 3.91)	< 0.001		15.9 (13.5, 18.8)	< 0.001

Note: ATT =  average treatment effect for the treated, representing participants observed in high-use trajectory groups; ATU =  average treatment effect for the untreated, representing participants observed in the low-use trajectory group; ATE =  average treatment effect in the full analytic sample.

### 4.5 ODUD symptoms

Membership in a high-use young adult polysubstance trajectory group was also associated with higher odds of ODUD symptoms in adulthood. In the ATT-weighted model, OR= 16.4; 95% CI: 13.7, 19.7; p < 0.001. In the ATU-weighted model, OR= 12.8; 95% CI: 8.42, 20.6; p < 0.001. In the ATE-weighted model, OR= 15.9; 95% CI: 13.5, 18.8; p < 0.001 (Table 2).

## 5 Discussion

In this national longitudinal cohort study, young adults with trajectories characterized by high use of alcohol, cannabis, and/or nicotine had much higher odds of later non-prescribed opioid use and ODUD symptoms between ages 35 and 55 than those in the low-use trajectory group. These associations were observed consistently across ATT, ATU, and ATE weighting approaches. Findings suggest that individuals with sustained high (versus low) use of at least one common substance (alcohol, cannabis, or cigarettes) during young adulthood were more likely to have later non-prescribed opioid use and other drug use disorder in adulthood.

These findings build on previous work connecting alcohol, cannabis, and nicotine use to non-prescribed opioid use and substance use disorder outcomes (Cicero et al., 2020, Compton et al., 2019, Crummy et al., 2020, Han et al., 2018, Han et al., 2017, Huang et al., 2019, Olfson et al., 2023, Votaw et al., 2019). Prior studies have documented associations between single substances and later opioid-related harms, as well as trajectories of prescription drug misuse and other substance use across adolescence and adulthood (Harton et al., 2023, Kwon et al., 2021, Lee et al., 2022, McCabe et al., 2022a). Related MTF trajectory work has also identified young adult polysubstance use trajectories and examined their association with non-prescribed opioid use during the same young adult developmental period (Harton and Parker, 2027). Using a nationally representative cohort, this study extends prior work by identifying distinct trajectories of alcohol, cannabis and nicotine use and examining their associations with adult outcomes decades later. In this study, elevated odds of later non-prescribed opioid use were observed across the broader set of high-use trajectory groups rather than being limited to one narrowly defined pattern of use. This suggests that risk may not be confined to one specific configuration of polysubstance use but might instead be associated with sustained high use of one or more common substances during young adulthood. Additionally, the association with ODUD symptoms was larger in magnitude than the association with non-prescribed opioid use, suggesting that early high-use trajectories could indicate vulnerability to a broader range of later drug-related consequences rather than to opioid use alone (Cicero et al., 2020, Compton et al., 2019, Crummy et al., 2020, McCabe et al., 2022c, McCabe et al., 2019b, McCabe et al., 2019a).

These results have several public health implications. Because alcohol, cannabis, and nicotine are common, legal, and/or widely accessible, and often addressed separately in prevention efforts, an exclusive focus on opioid-specific interventions could overlook earlier opportunities to reduce downstream harm. Screening and prevention strategies that identify persistent high-use patterns across multiple substances in young adulthood may help target individuals at elevated risk before later opioid-related and other drug-related harms emerge. It may also be beneficial to study interventions that are tailored to specific high-risk subsets of the population, particularly individuals with sustained high use of two or more substances during young adulthood (Cicero et al., 2020, Compton et al., 2019, Richter et al., 2017). Potential examples include developmentally appropriate screening and brief intervention approaches for alcohol, cannabis, nicotine, and nonmedical prescription drug use in primary care, college health, and community settings; brief alcohol interventions for adolescents and young adults; brief cannabis interventions for emerging adults; as well as integrated prevention approaches that address co-occurring use of commonly used substances rather than treating alcohol, cannabis, nicotine, and opioid-related risk as separate concerns (Cicero et al., 2020, Compton et al., 2019, Halladay et al., 2019, Richter et al., 2017, Tanner-Smith and Lipsey, 2015).

This study has several strengths. It used a large national longitudinal cohort with follow-up from age 18 into mid-adulthood, allowing examination of long term associations across a substantial portion of the life course (Patrick et al., 2022, Schulenberg et al., 2022). The analysis combined group-based trajectory modeling with propensity score weighting, which enabled identification of heterogeneous young adult substance use patterns and estimation of associations under a clearly specified measured confounding framework. The consistency of findings across ATT, ATU, and ATE estimands further strengthens confidence that the overall pattern was similar across weighting approaches.

This study also has some limitations. All substance use measures were based on self report and are therefore subject to recall error and misclassification. The exposure measures were dichotomized and did not capture intensity, frequency beyond any past-year use, or distinctions between experimental and more established patterns of substance use. The ODUD outcome reflects symptomology derived from survey items rather than clinical diagnoses and should not be interpreted as equivalent to opioid use disorder or any other specific diagnosed substance use disorder (American Psychiatric Association, 2013, McCabe et al., 2022c, Patrick et al., 2022, Schulenberg et al., 2022). In addition, the composite structure of the ODUD measure does not permit estimation of the prevalence of specific underlying substance use disorders, including opioid use disorder. Although propensity score weighting improved covariate balance across measured baseline characteristics, causal interpretation depends on assumptions that cannot be verified fully in observational data, including no unmeasured confounding, positivity and correct model specification (Rosenbaum and Rubin, 1983). In addition, although propensity score weighting improved balance on measured baseline covariates, residual and unmeasured confounding remain possible. Finally, the Monitoring the Future panel excludes some populations at particularly high risk of drug-related harm, including individuals not captured in school-based sampling at baseline, which limits generalizability (Patrick et al., 2022, Schulenberg et al., 2022).

Future research should examine whether specific combinations, timing and intensity of alcohol, cannabis, and nicotine use differentially shape later opioid-related risk, and whether these pathways vary across more recent cohorts exposed to changing policy and drug environments. Additional work using specific measures of opioid use disorder and other substance use disorders could also help clarify whether young adult high-use trajectories predict later diagnoses (Compton et al., 2019, McCabe et al., 2022c, Patrick et al., 2022).

In conclusion, trajectories characterized by high use of alcohol, cannabis and/or nicotine in young adulthood were associated with much higher odds of non-prescribed opioid use and ODUD symptoms in adulthood. Early polysubstance use patterns represent an important target for prevention, early intervention and harm reduction efforts aimed at reducing later opioid-related and other drug-related harms.

## CRediT authorship contribution statement

**Parker Maria A:** Writing – review & editing, Writing – original draft, Supervision, Conceptualization. **Zoh Roger:** Writing – review & editing, Methodology. **Dong-Chul Seo:** Writing – review & editing. **Jon Agley:** Writing – review & editing. **Harton Moriah:** Writing – review & editing, Writing – original draft, Formal analysis, Conceptualization.

## Contributors

Author Harton designed the study and conducted the statistical analysis. Authors Harton and Parker wrote the first draft of the manuscript. Authors Agley, Seo, and Zoh provided critical feedback. All authors contributed to and have approved the final manuscript.

## Declaration of Generative AI and AI-assisted technologies in the writing process

Generative AI was not used in the manuscript preparation process.

## Funding

Author Zoh was supported by 10.13039/100000062NIDDK Award # R01DK136994. The content is solely the responsibility of the authors and the US Government did not have a role in study design; collection, analysis, and interpretation of data; writing the report; or the decision to submit this report for publication.

This research did not receive any specific grant from funding agencies in the public, commercial, or not-for-profit sectors.

## Declaration of Competing Interest

The authors declare that they have no known competing financial interests or personal relationships that could have appeared to influence the work reported in this paper.
