# Supplementary material for: Young adult polysubstance use trajectories and later non-prescribed opioid use and drug use disorder symptoms: A propensity score-matched analysis
Source: Drug Alcohol Depend Rep. 2026 Sep 12;21:100479. doi: 10.1016/j.dadr.2026.100479 (PMC13597190; doi:10.1016/j.dadr.2026.100479)
Supplement: Supplementary file 1 [file mmc1.docx]

**Supplemental Figure 1.** Young adults' trajectories of cannabis, alcohol, and nicotine use from group-based trajectory models from baseline through the third follow-up in the MTF study. The columns represent probability of nicotine use (top row), alcohol (middle row), and cannabis use (bottom row) for each of the five groups (1–5, left to right) across the three follow-ups (x-axis). Data from the Monitoring the Future Study, 1976-2019.
