# Supplementary material for: Young adult polysubstance use trajectories and later non-prescribed opioid use and drug use disorder symptoms: A propensity score-matched analysis
Source: Drug Alcohol Depend Rep. 2026 Sep 12;21:100479. doi: 10.1016/j.dadr.2026.100479 (PMC13597190; doi:10.1016/j.dadr.2026.100479)
Supplement: Supplementary file 2 [file mmc2.docx]

**Supplemental Figure 2.** Weights from propensity score models for being in the high-use groups for the outcome of non-prescribed opioid use (left) and ODUD symptoms (right). Data from the Monitoring the Future Study, 1976-2019.
