# Supplementary material for: Young adult polysubstance use trajectories and later non-prescribed opioid use and drug use disorder symptoms: A propensity score-matched analysis
Source: Drug Alcohol Depend Rep. 2026 Sep 12;21:100479. doi: 10.1016/j.dadr.2026.100479 (PMC13597190; doi:10.1016/j.dadr.2026.100479)
Supplement: Supplementary file 3 [file mmc3.docx]

**Supplemental Table 1.** Monitoring the Future survey items, DSM-5 criterion mapping, and coding used to derive the other drug use disorder (ODUD) symptom indicator.

| **MTF Panel Question** | **DSM-V Criterion Mapping** | **Coding Scheme** |
| --- | --- | --- |
| Think back over the last five years. Did your use of alcohol, marijuana, or other illicit drugs  cause you any of the following problems? Hurt your performance on the job? | Substance use resulting in a failure to fulfill major role obligations | 0="No"  1="A little"  2="Some"  3="A lot"  If 1, 2, or 3, the answer was recoded as being TRUE  If 0, the answer was recoded as FALSE |
| Did your use of alcohol, marijuana, or other illicit drugs DURING THE LAST 5 YEARS lead to anyof the following? (For any drug that you did NOT use in the last 5 years, leave that column  blank.) You continued to use the drug even though you knew it was harmful to do so | Continued substance use when physically hazardous | 0="No"  1="A little"  2="Some"  3="A lot"  If 1, 2, or 3, the answer was recoded as being TRUE  If 0, the answer was recoded as FALSE |
| Think back over the last five years. Did your use of alcohol, marijuana, or other illicit drugs cause you any of the following problems? Caused you to behave in ways that you later regretted? | Continued substance use despite persistent or recurrent interpersonal or social problems; | 0="No"  1="A little"  2="Some"  3="A lot"  If 1, 2, or 3, the answer was recoded as being TRUE  If 0, the answer was recoded as FALSE |
| Did your use of alcohol, marijuana, or other illicit drugs DURING THE LAST 5 YEARS lead to any of the following? (For any drug that you did NOT use in the last 5 years, leave that column  blank.? Hurt your relationship with your child(ren)? | Continued substance use despite persistent or recurrent interpersonal or social problems; | 0="No"  1="A little"  2="Some"  3="A lot"  If 1, 2, or 3, the answer was recoded as being TRUE  If 0, the answer was recoded as FALSE |
| Think back over the last five years. Did your use of alcohol, marijuana, or other illicit drugs  cause you any of the following problems? Hurt your relationship with your parents? | Continued substance use despite persistent or recurrent interpersonal or social problems; | 0="No"  1="A little"  2="Some"  3="A lot"  If 1, 2, or 3, the answer was recoded as being TRUE  If 0, the answer was recoded as FALSE |
| Think back over the last five years. Did your use of alcohol, marijuana, or other illicit drugs  cause you any of the following problems? Hurt your relationship with your friends? | Continued substance use despite persistent or recurrent interpersonal or social problems; | 0="No"  1="A little"  2="Some"  3="A lot"  If 1, 2, or 3, the answer was recoded as being TRUE  If 0, the answer was recoded as FALSE |
| Did your use of alcohol, marijuana, or other illicit drugs DURING THE LAST 5 YEARS lead to any of the following? (For any drug that you did NOT use in the last 5 years, leave that column  blank.) You found that over time you needed more of the drug to get the same effect | Tolerance | 0="No"  1="A little"  2="Some"  3="A lot"  If 1, 2, or 3, the answer was recoded as being TRUE  If 0, the answer was recoded as FALSE |
| Did your use of alcohol, marijuana, or other illicit drugs DURING THE LAST 5 YEARS lead to anyof the following? (For any drug that you did NOT use in the last 5 years, leave that column  blank.) Stopping or reducing your use of the drug made you physically ill or sick  Did your use of alcohol, marijuana, or other illicit drugs DURING THE LAST 5 YEARS lead to anyof the following? (For any drug that you did NOT use in the last 5 years, leave that column  blank.) Stopping or reducing your use of the drug made you physically ill or sick | Withdrawal | 00="No"  1="A little"  2="Some"  3="A lot"  If 1, 2, or 3, the answer was recoded as being TRUE  If 0, the answer was recoded as FALSE |
| Did your use of alcohol, marijuana, or other illicit drugs DURING THE LAST 5 YEARS lead to anyof the following? (For any drug that you did NOT use in the last 5 years, leave that column  blank.) You used the drug to avoid "hangovers" or after-effects of the drug | Withdrawal | 0="No"  1="A little"  2="Some"  3="A lot"  If 1, 2, or 3, the answer was recoded as being TRUE  If 0, the answer was recoded as FALSE |
| Did your use of alcohol, marijuana, or other illicit drugs DURING THE LAST 5 YEARS lead to anyof the following? (For any drug that you did NOT use in the last 5 years, leave that column  blank.) You wanted to try to stop or cut down, but you found that you could not | Persistent desire or unsuccessful efforts to cut down substance use | 0="No"  1="A little"  2="Some"  3="A lot"  If 1, 2, or 3, the answer was recoded as being TRUE  If 0, the answer was recoded as FALSE |
| Think back over the last five years. Did your use of alcohol, marijuana, or other illicit drugs cause you any of the following problems? Caused you to be less stable emotionally? | Health-related issue(s) due to substance use | 0="No"  1="A little"  2="Some"  3="A lot"  If 1, 2, or 3, the answer was recoded as being TRUE  If 0, the answer was recoded as FALSE |
| Think back over the last five years. Did your use of alcohol, marijuana, or other illicit drugs cause you any of the following problems? Caused you to have less energy? | Health-related issue(s) due to substance use | 0="No"  1="A little"  2="Some"  3="A lot"  If 1, 2, or 3, the answer was recoded as being TRUE  If 0, the answer was recoded as FALSE |
| Think back over the last five years. Did your use of alcohol, marijuana, or other illicit drugs cause you any of the following problems? Made you feel bad (e.g., depressed, anxious, ashamed) for more than just a few days? | Health-related issue(s) due to substance use | 0="No"  1="A little"  2="Some"  3="A lot"  If 1, 2, or 3, the answer was recoded as being TRUE  If 0, the answer was recoded as FALSE |
| Think back over the last five years. Did your use of alcohol, marijuana, or other illicit drugs cause you any of the following problems? Caused your physical health to be bad? | Health-related issue(s) due to substance use | 0="No"  1="A little"  2="Some"  3="A lot"  If 1, 2, or 3, the answer was recoded as being TRUE  If 0, the answer was recoded as FALSE |
| Did your use of alcohol, marijuana, or other illicit drugs DURING THE LAST 5 YEARS lead to anyof the following? (For any drug that you did NOT use in the last 5 years, leave that column  blank.) You felt such a strong desire to use the drug that you could not resist it or think of  anything else | Craving | 0="No"  1="A little"  2="Some"  3="A lot"  If 1, 2, or 3, the answer was recoded as being TRUE  If 0, the answer was recoded as FALSE |
