# Supplementary material for: Young adult polysubstance use trajectories and later non-prescribed opioid use and drug use disorder symptoms: A propensity score-matched analysis
Source: Drug Alcohol Depend Rep. 2026 Sep 12;21:100479. doi: 10.1016/j.dadr.2026.100479 (PMC13597190; doi:10.1016/j.dadr.2026.100479)
Supplement: Supplementary file 4 [file mmc4.docx]

**Supplemental Table 2.** Propensity score model for membership in the high-use groups. Data from the Monitoring the Future Study, 1976-2019.

|  | Non-Prescribed Opioid Use | | ODUD Symptoms | |
| --- | --- | --- | --- | --- |
|  | Odds Ratio (95% CI) | p-value | Odds Ratio (95% CI) | p-value |
| *Sex* |  |  |  |  |
| Male | — |  | — |  |
| Female | 0.93 (0.86, 1.00) | 0.047 | 0.92 (0.85, 0.99) | 0.033 |
| *Race* |  |  |  |  |
| White | — |  | — |  |
| Black | 0.36 (0.32, 0.40) | <0.001 | 0.36 (0.32, 0.41) | <0.001 |
| Asian | 0.40 (0.33, 0.49) | <0.001 | 0.40 (0.33, 0.50) | <0.001 |
| Hispanic | 0.69 (0.59, 0.82) | <0.001 | 0.69 (0.59, 0.82) | <0.001 |
| Other | 0.57 (0.47, 0.69) | <0.001 | 0.58 (0.48, 0.71) | <0.001 |
| Unknown | 0.63 (0.44, 0.94) | 0.02 | 0.67 (0.46, 1.01) | 0.045 |
| *Urbanicity* |  |  |  |  |
| Small Town | — |  | — |  |
| Rural | 0.74 (0.67, 0.82) | <0.001 | 0.74 (0.66, 0.82) | <0.001 |
| Suburban | 1.25 (1.11, 1.40) | <0.001 | 1.24 (1.10, 1.39) | <0.001 |
| Urban | 1.17 (1.05, 1.29) | 0.004 | 1.17 (1.05, 1.30) | 0.005 |
| Unknown | 0.87 (0.75, 1.01) | 0.062 | 0.88 (0.75, 1.02) | 0.089 |
| *Census Region* |  |  |  |  |
| South | — |  | — |  |
| Northeast | 2.59 (2.30, 2.92) | <0.001 | 2.58 (2.28, 2.91) | <0.001 |
| Midwest | 1.84 (1.67, 2.03) | <0.001 | 1.87 (1.70, 2.06) | <0.001 |
| West | 0.92 (0.83, 1.02) | 0.1 | 0.93 (0.84, 1.03) | 0.2 |
| *Mother’s Highest Educ.* |  |  |  |  |
| High School Grad | — |  | — |  |
| Less than High School | 1.00 (0.89, 1.12) | >0.9 | 1.00 (0.89, 1.12) | >0.9 |
| Some College | 1.06 (0.95, 1.18) | 0.3 | 1.06 (0.95, 1.18) | 0.3 |
| College Grad | 1.09 (0.98, 1.21) | 0.11 | 1.08 (0.97, 1.21) | 0.2 |
| Grad School | 1.33 (1.15, 1.54) | <0.001 | 1.36 (1.17, 1.58) | <0.001 |
| Unknown | 0.76 (0.63, 0.92) | 0.004 | 0.76 (0.63, 0.92) | 0.005 |
| *Num. Parents in Home* |  |  |  |  |
| 2 | — |  | — |  |
| 1 | 1.12 (1.02, 1.25) | 0.025 | 1.14 (1.03, 1.27) | 0.013 |
| 0/Unknown | 1.17 (0.98, 1.42) | 0.089 | 1.20 (0.99, 1.45) | 0.062 |
| *GPA* |  |  |  |  |
| B | — |  | — |  |
| A | 0.57 (0.53, 0.62) | <0.001 | 0.57 (0.53, 0.62) | <0.001 |
| C/D/Unknown | 1.33 (1.19, 1.48) | <0.001 | 1.31 (1.18, 1.46) | <0.001 |
|  |  |  |  |  |
